# PKCε phosphorylation regulates the mitochondrial translocation of ATF2 in ischemia-induced neurodegeneration

**DOI:** 10.1186/s12868-018-0479-z

**Published:** 2018-11-29

**Authors:** Varun Kumar, Yi-Chinn Weng, Yu-Chieh Wu, Yu-Ting Huang, Wen-Hai Chou

**Affiliations:** 1 Department of Biological Sciences, School of Biomedical Sciences, Kent State University, Kent, OH 44242 USA; 2 Center for Neuropsychiatric Research, National Health Research Institutes, Miaoli, 35053 Taiwan, ROC

**Keywords:** PKC, ATF2, Global cerebral ischemia, Neurodegeneration

## Abstract

**Background:**

Global cerebral ischemia triggers neurodegeneration in the hippocampal CA1 region, but the mechanism of neuronal death remains elusive. The epsilon isoform of protein kinase C (PKCε) has recently been identified as a master switch that controls the nucleocytoplasmic trafficking of ATF2 and the survival of melanoma cells. It is of interest to assess the role of PKCε–ATF2 signaling in neurodegeneration.

**Results:**

Phosphorylation of ATF2 at Thr-52 was reduced in the hippocampus of PKCε null mice, suggesting that ATF2 is a phosphorylation substrate of PKCε. PKCε protein concentrations were significantly reduced 4, 24, 48 and 72 h after transient global cerebral ischemia, resulting in translocation of nuclear ATF2 to the mitochondria. Degenerating neurons staining positively with Fluoro-Jade C exhibited cytoplasmic ATF2.

**Conclusions:**

Our results support the hypothesis that PKCε regulates phosphorylation and nuclear sequestration of ATF2 in hippocampal neurons during ischemia-induced neurodegeneration.

## Background

Cardiac arrest is a leading cause of death and disability worldwide [1, 2]. Global cerebral ischemia resulting from cardiac arrest causes selective neurodegeneration in the hippocampal CA1 region [3–6].

Previous studies have shown that pharmacological activation of PKCε induces ischemic tolerance [7]. PKCε peptide activator (ψεRACK) administered before ischemia protects neurons from ischemic insult [8–11]. The beneficial effects of PKCε peptide activators in ischemic preconditioning prompted our interest in understanding the molecular and cellular activity of PKCε in disease progression [7].

A detailed understanding of PKCε signaling pathways requires identification of its unique targets, one of which is activating transcription factor 2 (ATF2). Recent studies have found that ATF2 is a phosphorylation substrate of PKCε in melanoma cells [12–14], but it is unknown whether this phosphorylation occurs in neurons. ATF2, a member of the activator protein 1 (AP1) transcription factor superfamily, regulates normal growth and development and response to cellular stress [13, 15]. PKCε phosphorylates ATF2 at Thr-52, which is required for nuclear retention of ATF2. In response to genotoxic stress, PKCε activity is decreased [12–14]. This leads to mitochondrial translocation of ATF2 and subsequent apoptosis in melanoma cells.

In the present study, we identified ATF2 as a PKCε phosphorylation substrate in the hippocampus. Reduction of PKCε after tGCI correlated temporally with mitochondrial translocation of ATF2 and degeneration of hippocampal neurons. Our data support the hypothesis that PKCε phosphorylation regulates ATF2 subcellular translocation and neurodegeneration after cerebral ischemia.

## Methods

### Transient global cerebral ischemia (tGCI)

PKCε null mice generated by homologous recombination were generous gifts from Dr. Robert Messing at the University of Texas Austin [16, 17]. Male and female PKCε heterozygous mice maintained on C57BL/6J and 129S4 backgrounds were crossed to produce F1 hybrid mice. The F1 hybrid mice were intercrossed to generate F2 hybrid littermates for experiments. Mice were genotyped via PCR of DNA extracted from mouse tails. Male F2 hybrid wild-type (WT) and PKCε null mice between 2 and 4 months of age were used for all experiments [16, 17].

Total 105 mice were subjected to tGCI and randomly assigned into different groups of analysis (3 WT and 3 PKCε null mice for PKCε phosphorylation of ATF2 at T52, 29 WT mice for PKCε expression in the hippocampus after tGCI, 43 WT mice for temporal expression of ATF2 in the hippocampal mitochondria after tGCI, 12 WT mice for histological analysis of the CA1 region of the hippocampus after tGCI, 9 WT mice for mitochondrial translocation of ATF2 after tGCI, 6 WT mice for expression of ATF2 in degenerating neurons labeled with Fluoro-Jade C).

Before tGCI, mice were fasted overnight but allowed free access to water. Mice were anesthetized with 5% isoflurane in a mixture of N_2_O/O_2_ (70%/30%) and maintained on 1.5% isoflurane using the MouseVent G500 Automatic Ventilator (Kent Scientific, Torrington, CT, USA). TGCI was induced by hypotension and occlusion of bilateral common carotid arteries using microclips for 10 min, following a previously-described procedure [4]. Control mice were sham-operated under the same surgical conditions.

All mouse-related experiments were conducted in compliance with the guidelines of the Office of Research Compliance at Kent State University and the Laboratory Animal Center of National Health Research Institutes. The animal protocols were approved by Research Involving Animal (Institutional Animal Care and Use Committee, IACUC) under Office of Research Compliance at Kent State University (Protocol# 359 CW 14-8 rev 3 Sub 27 August 2013) and the Laboratory Animal Center at National Health Research Institutes (Protocol# NHRI-IACUC-105087-A), according to the Guide for the Care and Use of Laboratory Animals (NRC 2011). Management of animal experiment and animal care and use of Kent State University and National Health Research Institutes have accredited by the AAALAC. All efforts were made to minimize the number of animals used.

### Western blot analysis

At different time points after tGCI, mice were deeply anesthetized with 4% isoflurane in N_2_O/O_2_ (70%/30%) and euthanized by cervical dislocation. Mouse hippocampi were isolated at different time points after tGCI and homogenized using a Teflon-glass homogenizer as previously described [17]. Mitochondrial and cytosolic fractions were prepared using a mitochondrial isolation kit (Thermo Scientific Pierce, Rockford, lL, USA). The whole cell lysates, mitochondrial and cytosolic fractions were analyzed by Western blot using rabbit anti-Thr-52 ATF2 (1:200, Phosphosolution, Aurora, CO, USA, Cat# p115-52), rabbit anti-ATF2 (1:1000, Cell Signaling Technology, Danvers, MA, USA, Cat# 9226), mouse anti-Actin (1:1000, Sigma-Aldrich, St. Louis, MO, USA, Cat# A4700), mouse anti-PKCε (1:1000, BD, Franklin Lakes, NJ, USA, Cat# 610086) and rabbit anti-COXIV (1:1000, Sigma-Aldrich, Cat# AV42784). Immunoreactive bands were detected by enhanced chemiluminescence (ECL) (Thermo Scientific Pierce, Rockford, lL, USA), imaged by Luminescent Image Analyzer LAS-3000 (Fujifilm, Edison, NJ, USA) and quantified by scanning densitometry using the NIH ImageJ program.

### Hippocampal neuronal culture and oxygen–glucose–deprivation

Primary neuronal cultures were prepared from the hippocampus of postnatal day 1 (P1) to P3 mice as previously described [17, 18]. The cultures were subjected to 1 h of oxygen–glucose–deprivation and 4 h of re-oxygenation to simulate transient ischemia [18]. To label mitochondria, cultures were incubated with 100 nM MitoTracker for one hour (Thermo Scientific Invitrogen, Carlsbad, CA, USA), fixed with 4% paraformaldehyde (PFA), and immunostained with an antibody against ATF2 (1:200, Sigma-Aldrich, Cat#SAB4300315) and DAPI as previously described [18]. The images were obtained using an Olympus (Center Valley, PA, USA) FV500/IX81 confocal microscope.

### Histology

Mice were deeply anesthetized with 4% isoflurane in N_2_O/O_2_ (70%/30%) at different time points after tGCI and perfused transcardially with 4% PFA in 0.15 M phosphate buffer (pH 7.3) [18]. Brains were isolated and postfixed in 4% PFA. Coronal Sects. (1 mm) were obtained using a brain matrix (Braintree Scientific, Braintree, MA, USA) and processed for paraffin embedding. Coronal Sects. (5 μm) from the hippocampus (-3.5 mm caudal to bregma) were obtained using a microtome. Paraffin sections mounted on slides were stained with hematoxylin and eosin (H&E).

### Immunofluorescence staining

Primary neurons and paraffin brain Sects. (5 μm) were fixed with 4% PFA and incubated overnight at 4 °C with rabbit anti-ATF2 (1:200, Cell Signaling Technology, Cat# 9226) and mouse anti-COXIV (1:200, Cell Signaling Technology, Cat#11967) [17, 18]. Degenerating neurons were detected using a Fluoro-Jade C staining kit (Histo-Chem, Jefferson, AR, USA) [19]. After washing, the cultured neurons and brain sections were stained with Alexa Fluor conjugated secondary antibodies (1:200, Jackson ImmunoResearch, West Grove, PA, USA) and mounted in media containing 4′, 6-diamidino-2-phenylindole (DAPI) (Vector Laboratories, Burlingame, CA, USA). The images were acquired using an Olympus FV500/IX81 confocal microscope or a Leica TCS SP5 II confocal laser scanning microscope.

### Statistical analysis

Quantitative data are mean ± SEM *t* test, one-way ANOVA, and Newman-Keuls post hoc tests were used for statistical analysis. Values of *P*< 0.05 in all tests were considered to be statistically significant.

## Results

### PKCε phosphorylation of ATF2 at Thr-52 in hippocampus

**Fig. 1:**
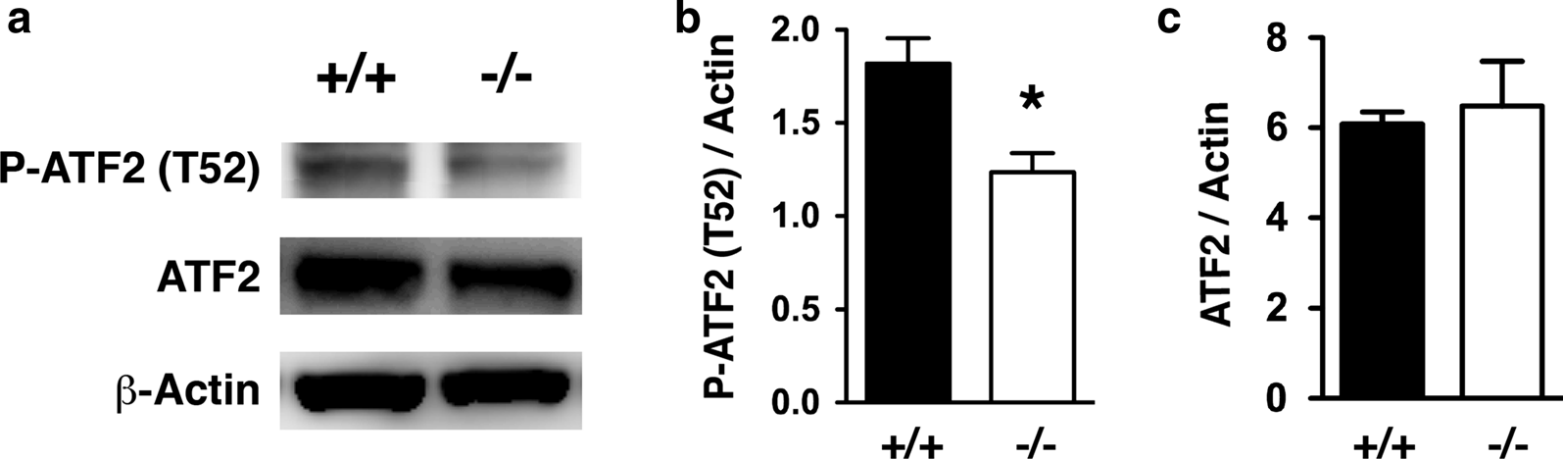
PKCε phosphorylation of ATF2 at T52 in vivo. **a** Representative Western blot showing that anti-phospho-ATF2 (T52) antibody detected less immunoreactivity in hippocampal lysates from *Prkce*^−*/*−^ than *Prkce*^+*/*+^ mice. **b** The ratio of phospho-ATF2 (T52) to β-Actin immunoreactivity was significantly reduced in the lysates from *Prkce*^−*/*−^ (*n* = 3) as compared to *Prkce*^+*/*+^ mice (*n* = 3) (*P < 0.05, two-tailed, unpaired *t*-test). **c** The ratio of ATF2 to β-Actin immunoreactivity was similar between *Prkce*^+*/*+^ (*n* = 3) and *Prkce*^−*/*−^ mice (*n* = 3). Error bars show SEM

Phospho-ATF2 (T52) immunereactivity was reduced in PKCε null mice, whereas ATF2 and Actin immunoreactivity were similar between WT and PKCε null mice (Fig. 1a). The ratio of phospho-ATF2 (T52) to Actin immunoreactivity was significantly reduced in the hippocampal lysates from PKCε null mice as compared to WT mice, suggesting that PKCε phosphorylates ATF2 at Thr52 in the hippocampus (Fig. 1b). On the other hand, the ratio of ATF2 to Actin was not altered in PKCε null mice, suggesting that PKCε was not involved in regulating the expression of ATF2 (Fig. 1c).

### Progressive reduction of PKCε expression after tGCI

**Fig. 2:**
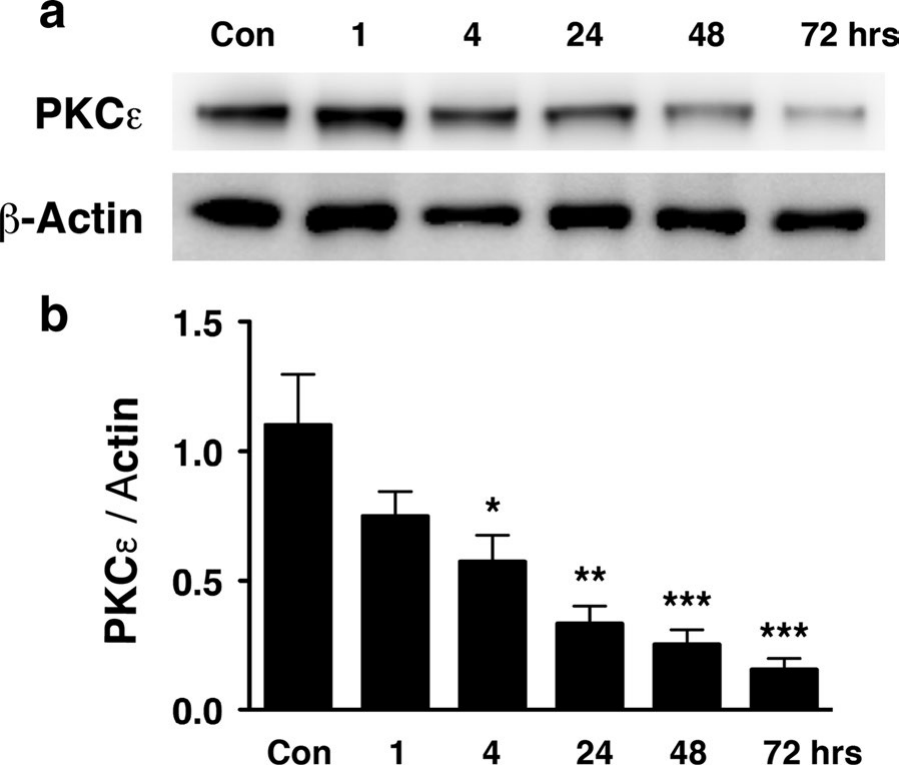
PKCε expression was gradually reduced in the hippocampus after tGCI. **a** The mouse hippocampus was isolated at different time points after 10 min of tGCI. Hippocampi of mice without tGCI were used as controls (con). Cytosolic fractions of hippocampus were analyzed by Western blotting using antibodies against PKCε. β-Actin was used as a loading control. **b** PKCε and β-Actin protein bands were quantified by densitometry (*n* = 4–7). PKCε immunoreactivity normalized to β-Actin (PKCε/β-Actin) was compared between means using one-way ANOVA with Newman**–**Keuls post hoc tests. There were significant reductions of PKCε/β-Actin at 4 (*P < 0.05), 24 (**P < 0.005), 48 and 72 h (***P < 0.0005) after tGCI as compared to controls

PKCε immunoreactivity was gradually reduced in the hippocampus after tGCI (Fig. 2a). The ratio of PKCε to Actin immunoreactivity was significantly reduced at 4, 24, 48 and 72 h after tGCI, as compared with non-ischemic controls (Fig. 2b).

### Mitochondrial translocation of ATF2 after tGCI

**Fig. 3:**
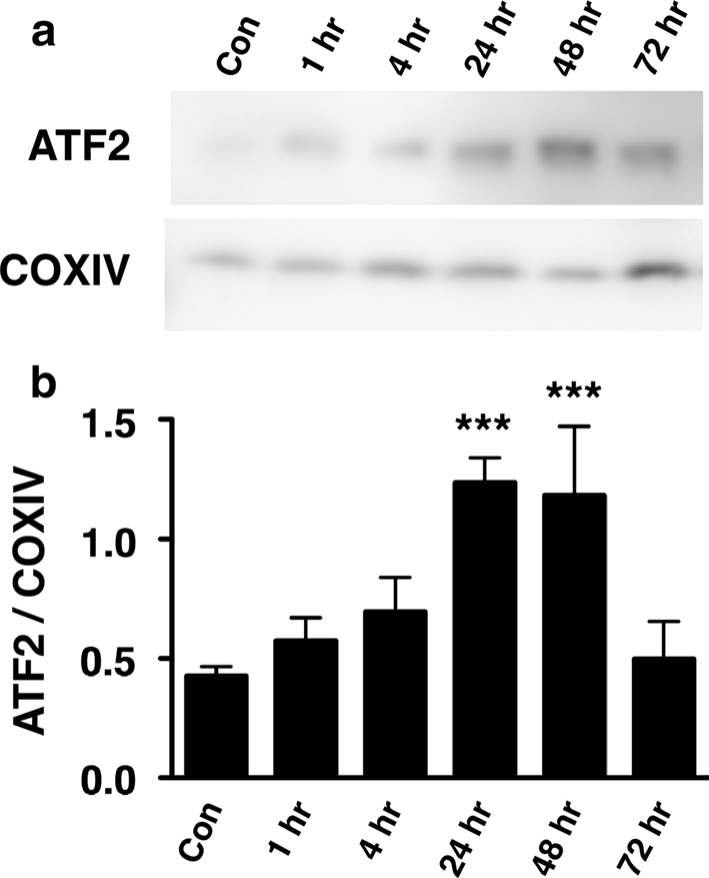
Temporal expression of ATF2 in the hippocampal mitochondria after tGCI. **a** Mitochondrial fractions of hippocampus isolated at different time points after tGCI were analyzed by Western blotting using antibodies against ATF2. COXIV was used as a loading control for the mitochondria. **b** Quantitative analysis of ATF2/COXIV protein bands by densitometry demonstrated a significant induction of mitochondrial ATF2 expression 24 and 48 h after tGCI as compared with controls (*n *= 4–5) (***p < 0.0005, one-way ANOVA with Newman-Keuls post hoc tests)

ATF2 immunoreactivity was gradually increased in the mitochondrial fraction of hippocampus after tGCI (Fig. 3a). The ratio of ATF2 to COXIV immunoreactivity was significantly increased at 24 and 48 h after tGCI as compared to non-ischemic controls (Fig. 3b). Mitochondrial translocation of ATF2 correlates temporally with the significant reduction of PKCε starting 4 h after tGCI (Fig. 2). This finding suggests that ATF2 was released from nucleus to the mitochondria in response to the reduction of PKCε after tGCI.

**Fig. 4:**
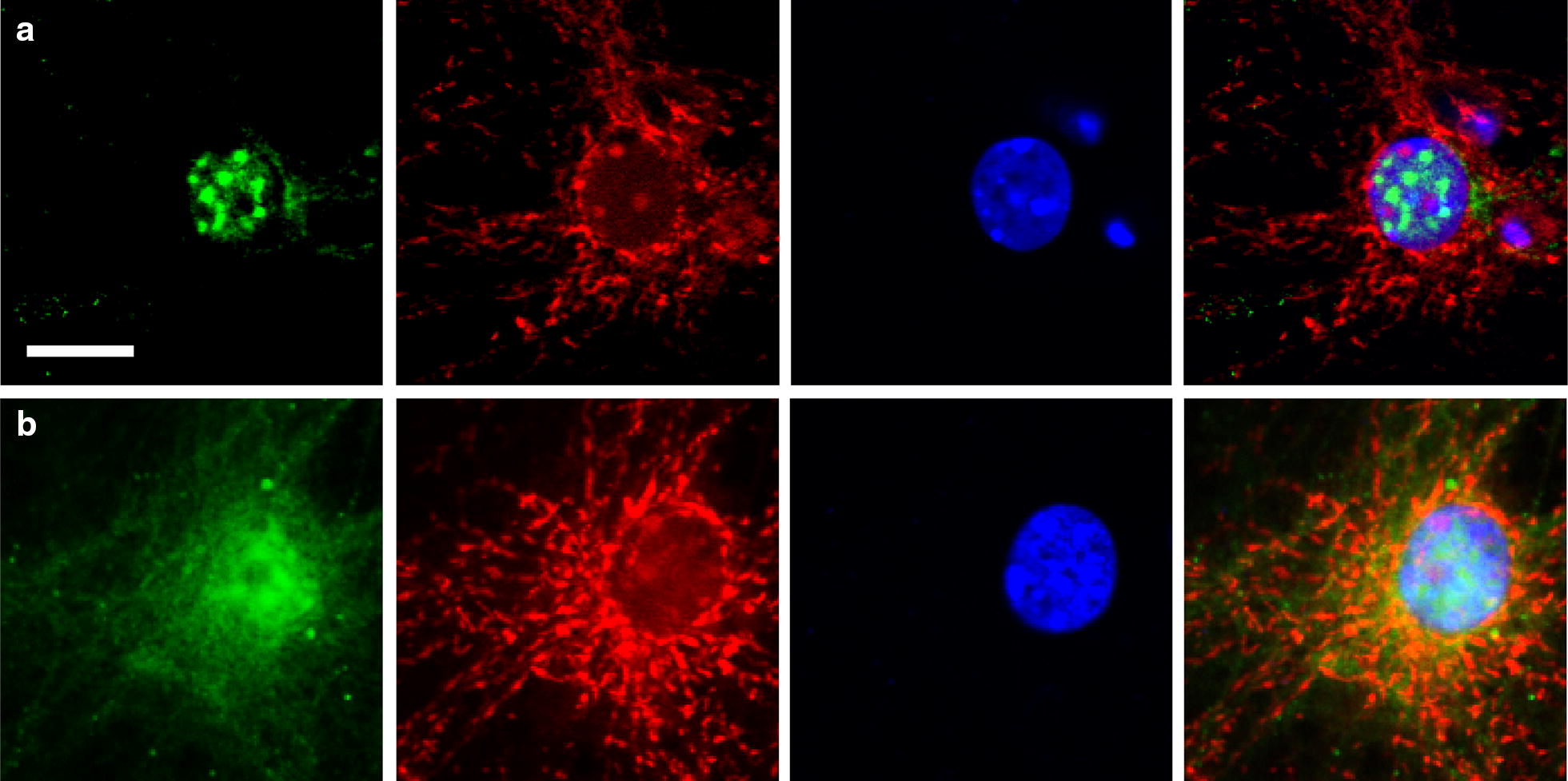
Mitochondrial translocation of ATF2 after oxygen–glucose–deprivation. Confocal images show the expression pattern of ATF2 (green) and MitoTracker (red) in cultured hippocampal neurons under non-ischemic conditions (**a**) and 4 h after oxygen–glucose–deprivation (**b**). Nuclei were stained with DAPI (blue). Scale bars, 10 μm

Neuronal cultures incubated with MitoTracker, a cell-permeant mitochondrial labeling dye, were used to visualize subcellular translocation of ATF2. We then performed immunofluorescence staining using antibodies against ATF2. Under non-ischemic conditions, ATF2 was detected exclusively in the nucleus (Fig. 4a). ATF2 expression became dispersed and co-localized with mitochondria after one hour of oxygen–glucose–deprivation and 4 h of reoxygenation (Fig. 4b).

**Fig. 5:**
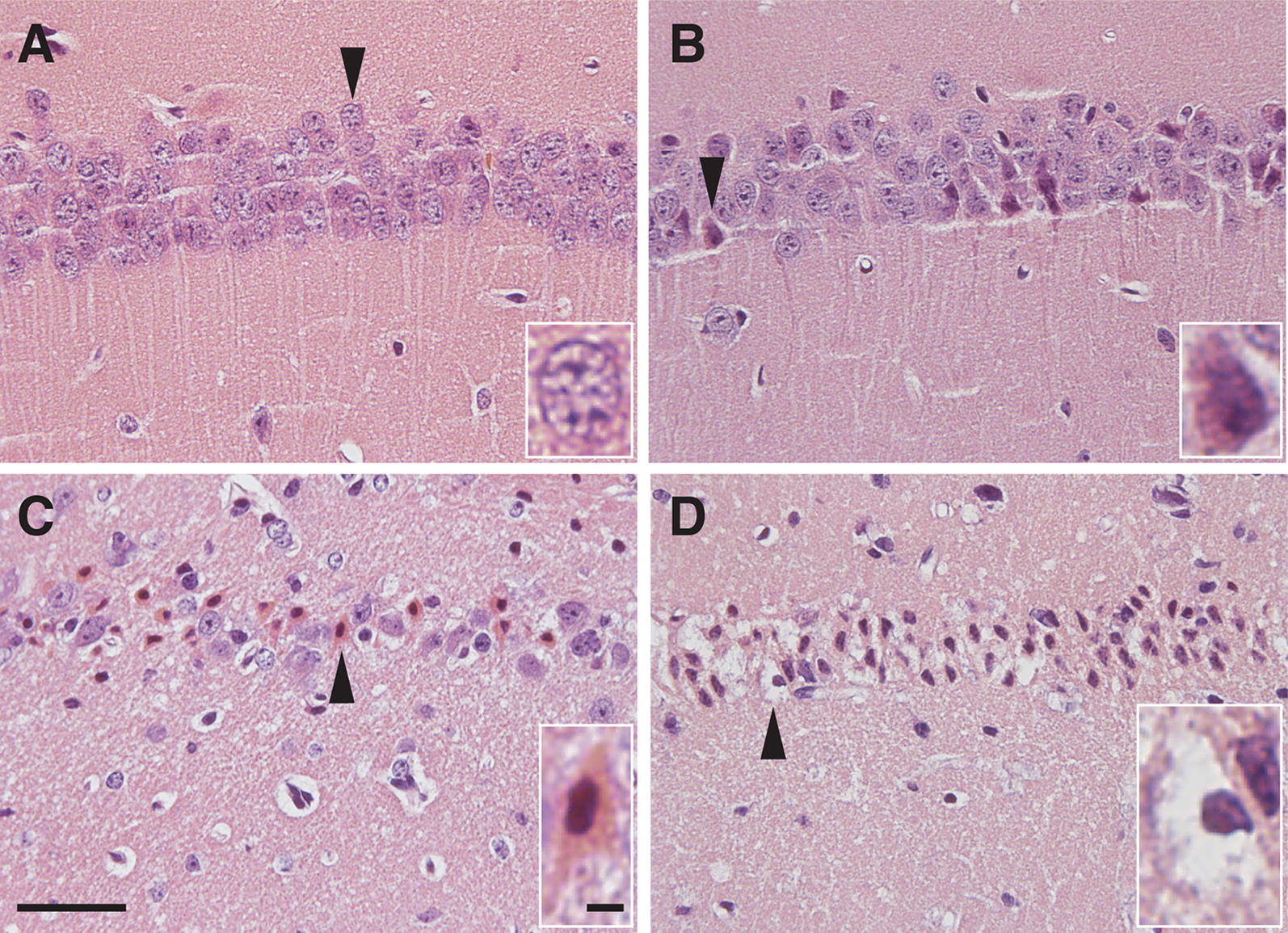
Histological analysis of the CA1 region of the hippocampus after tGCI. Representative images of H&E stained brain sections prepared from non-ischemic control mice (**a**), and 24 h (**b**), 48 h (**c**) and 72 h (**d**) after tGCI. CA1 pyramidal neurons of control mice show large, round and pale-stained nuclei (**a**). Ischemic eosinophilia (pink color) was detected in the cytoplasm and dendrites of degenerating neurons 24 and 48 h after tGCI (arrowheads in **b** and **c**). Neurons with both eosinophilic cytoplasm and pyknotic nuclei were detected 48 h after tGCI (**c**). Degenerative neurons showing pyknotic nuclei and empty cytoplasm were detected 48 and 72 h after tGCI (arrowheads in **d**). Representative neurons (arrowhead) are shown amplified in the lower right side of each panel. Scale bars = 40 μm for main images and scale bars = 4 μm for the magnified images

Neurons undergoing ischemic neurodegeneration exhibit hyper-eosinophilic cytoplasm and dark pyknotic nuclei using H&E staining [3–6]. The earliest morphological changes appeared in the CA1 region 24 h after tGCI (Fig. 5). Some CA1 pyramidal neurons started to show eosinophilia (acidophilic, pink) in the cytoplasm 24 h after tGCI (Fig. 5b). The number of neurons with eosinophilic cytoplasm increased further at 48 h (Fig. 5c). These eosinophilic neurons also exhibited condensation of chromatin in the nucleus (pyknosis). The cytoplasm of most pyknotic neurons became empty by 72 h after tGCI (Fig. 5d).

**Fig. 6:**
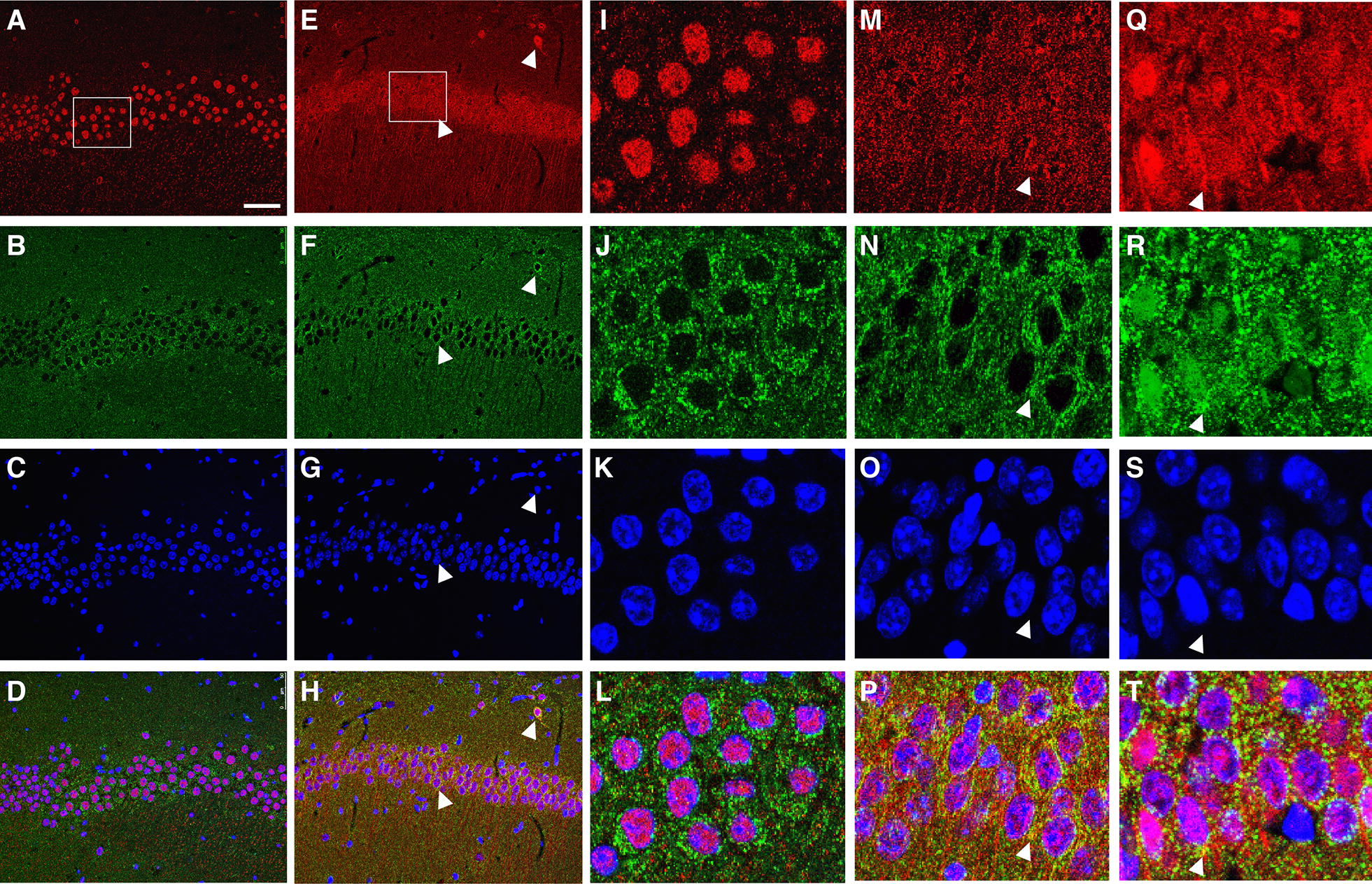
Mitochondrial translocation of ATF2 after tGCI. Confocal images of ATF2 (red) and COXIV (green) in the CA1 of the hippocampus under non-ischemic conditions (**a**–**d**, **i**–**l**), 24 (**e**–**h**, **m**–**p**) and 48 h (**q**–**t**) after tGCI. Amplified images are shown in the right panels (**i**–**t**). Merged images (**d**, **h**, **l**, **p**, **t**) indicate colocalization of ATF2 with COXIV (yellow) after tGCI (white arrowheads). Scale bars, 50 μm

To reveal the subcellular translocation of ATF2 in intact hippocampus, mouse brain sections collected at different time points after tGCI were processed for immunofluorescence staining. Similar to the cultured neurons (Fig. 4), ATF2 immunoreactivity was detected in the nucleus under non-ischemic conditions (Fig. 6a–d and i–l). ATF2 immunoreactivity was detected outside of the nucleus and colocalized with mitochondria labeled with COXIV at 24 (Fig. 6e–h, m–p) and 48 h (Fig. 6q–t) after tGCI.

**Fig. 7:**
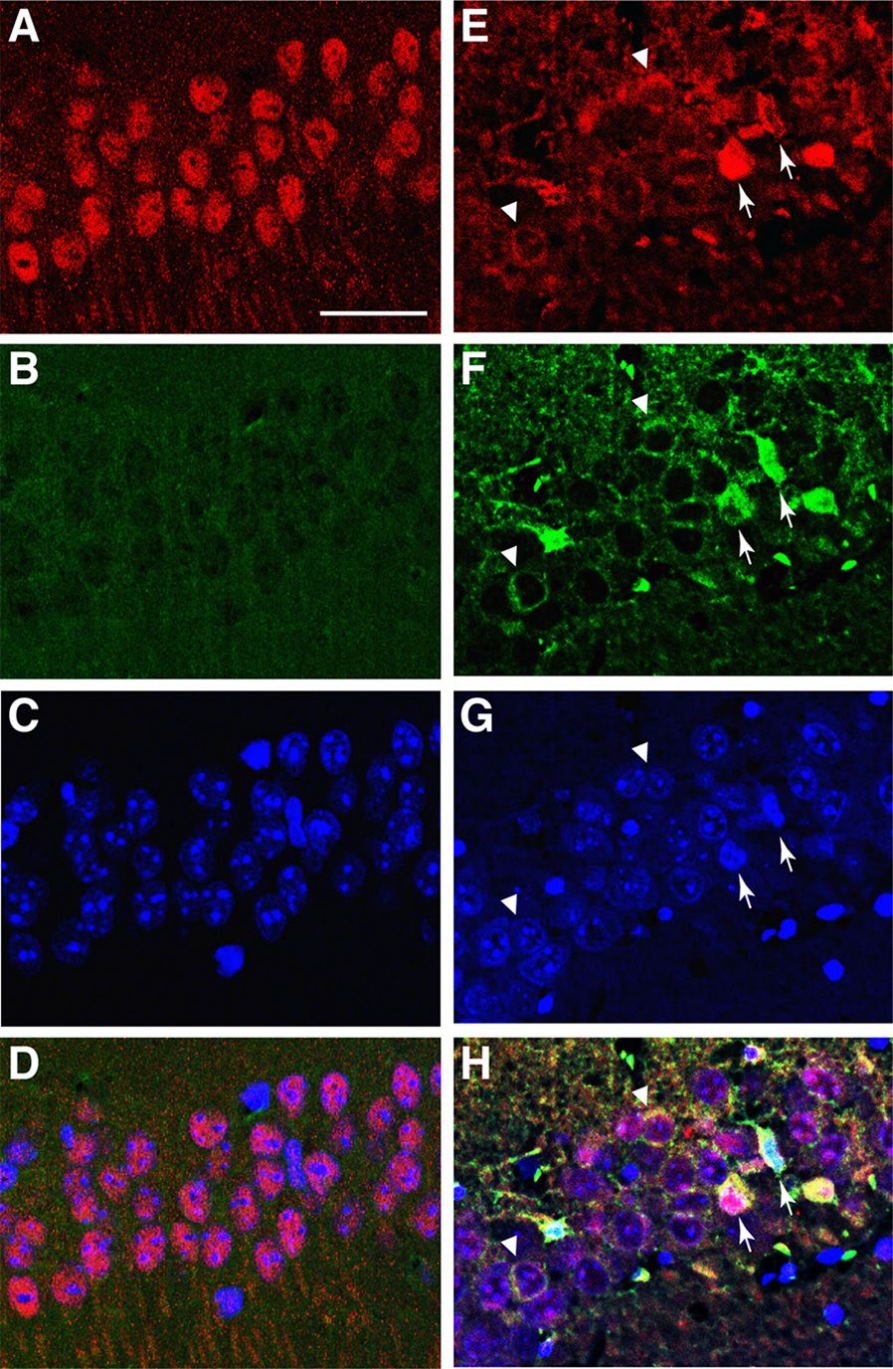
Expression of ATF2 in degenerating neurons labeled with Fluoro-Jade C. Confocal images of ATF2 (red) and Fluoro-Jade C (green) in the CA1 of the hippocampus under non-ischemic conditions (**a**–**d**) and 48 h after tGCI (**e**–**h**). Merged images (**d**, **h**) indicate colocalization of ATF2 with Fluoro-Jade C (yellow) after tGCI (**h**). Neurons with cytoplasmic expression of ATF2 (arrowheads) and induced expression of ATF2 (arrows) were indicated in panel E-H. Scale bars, 25 μm

To assess whether the subcellular localization of ATF2 is associated with neurodegeneration, mouse brain sections prepared 48 h after tGCI were stained with anti-ATF2 antibody and Fluoro-Jade C. Degenerating neurons with intact nuclei were lightly stained with Fluoro-Jade C and exhibited cytoplasmic ATF2 expression (Fig. 7). Degenerative neurons with condensed pyknotic nuclei exhibited intense induction of ATF2 in the cytoplasm and intense Fluoro-Jade C staining. Immunohistochemical detection of ATF2 protein (Fig. 4, 5, 6, 7) supports the Western blot finding that ATF2 was gradually exported from nucleus to the mitochondria of hippocampal neurons in response to cerebral ischemia (Fig. 3).

## Discussion

**Fig. 8:**
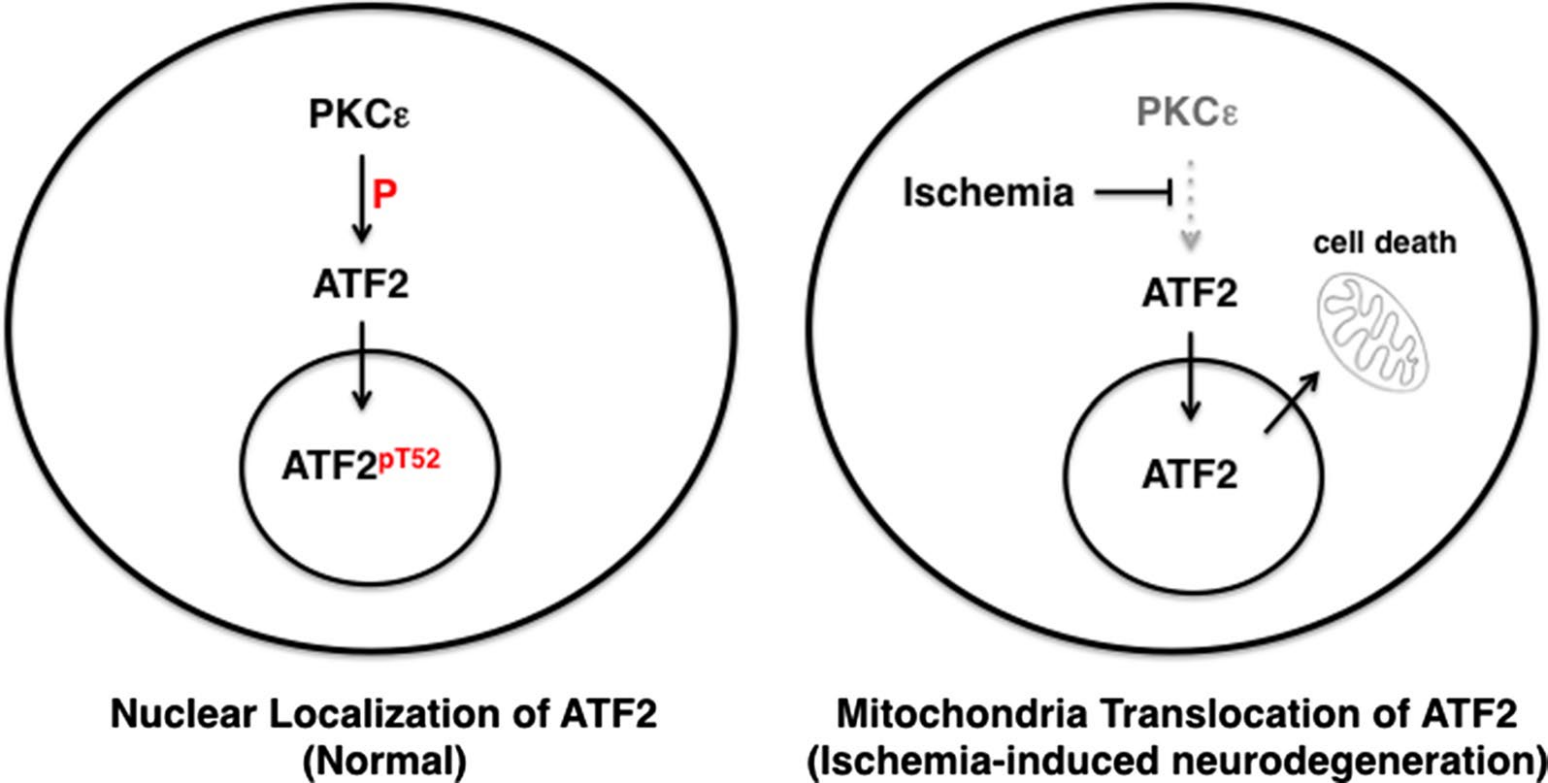
A proposed model for PKCε phosphorylation of ATF2 during ischemia-induced neurodegeneration. Phosphorylation of ATF2 by PKCε at Thr-52 is required for the nuclear localization of ATF2. The expression level of PKCε is attenuated in response to global cerebral ischemia, resulting in translocation of ATF2 to the mitochondria and degeneration of neuronal cells

Our results suggest that the PKCε–ATF2 signaling pathway is involved in the regulation of neurodegeneration in the hippocampus after cerebral ischemia (Fig. 8). We found that PKCε phosphorylates ATF2 at Thr-52 in hippocampal neurons. In response to global cerebral ischemia, the expression of PKCε was gradually reduced, resulting in mitochondrial translocation of ATF2 and neurodegeneration. Although ATF2 has previously been reported to be a PKCε phosphorylation substrate in melanoma cells, in neuronal cells ATF2 phosphorylation by PKCε has not previously been studied. We have also characterized the regulatory role of PKCε in nucleocytoplasmic trafficking of ATF2 after global cerebral ischemia.

PKCε peptide activator treatment before cerebral ischemia confers neuroprotection [8–11], but treatments after ischemia show no effect [11, 20]. The present findings suggest that the loss of the protective effect of PKCε peptide activator after ischemia may be due to reduced expression of PKCε; i.e. a lack of PKCε available to be activated (Fig. 2).

Although ATF2 is generally localized to the nucleus where it functions as a transcription factor, it has been detected in the cytoplasm of degenerating neurons of patients with Alzheimer’s, Parkinson’s and Huntington’s diseases [21, 22]. Nerve injury caused by nerve fiber transection or doxorubicin is connected to the reduction of ATF2 in the nucleus [23]. These reports suggest cytoplasmic localization of ATF2 following stress or damage is associated with neuronal cell death. In the squamous carcinoma cell line SCC-9, ATF2 translocates to the mitochondria following genotoxic stress and disrupts the Hexokinase 1 (HK1)—Voltage dependent anion channel 1 (VDAC1) complex, thereby compromising mitochondrial membrane pore permeability and promoting apoptosis [12–14]. This raises the intriguing possibility that a similar mechanism of ATF2-HK1-VDAC1 interaction is present in degenerating neurons. The present findings not only connect cytoplasmic ATF2 with ischemic neurodegeneration, but also suggest that PKCε is a master switch regulating neuronal survival and degeneration in various types of neurodegenerative diseases.

The nuclear export of ATF2 is mediated by Exportin 1 (XPO1), also known as chromosome region maintenance protein 1 (CRM1) [12–14, 24, 25]. XPO1 is a member of the Karyopherin β family that recognizes proteins containing hydrophobic leucine-rich nuclear export sequences, and transports them from nucleus to the cytoplasm [12–14, 24, 25]. Leptomycin B (LMB), an anti-fungal antibiotic, has been shown to inhibit nuclear export pathways mediated by XPO1 [24, 25]. Treatment with LMB inhibits nuclear export of ATF2 in melanoma cells [12–14]. XPO1 is up-regulated in neurons in response to traumatic brain injury [26], multiple sclerosis [27, 28] and cerebral ischemia [29], suggesting that the progression of these neurological disorders may in part be mediated by exaggerated nuclear export. Thus, inhibition of XPO1 may prove useful in reducing neurodegeneration in these neurological disorders, including cerebral ischemia.

## Conclusions

In summary, we used both biochemical and histochemical approaches to examine the regulatory role of PKCε after global cerebral ischemia. Our results suggest that the presence of PKCε is essential for Thr-52 phosphorylation and the nucleocytoplasmic trafficking of ATF2 in the hippocampus. This study shows that mitochondrial translocation of ATF2 is associated with neurodegeneration after global cerebral ischemia.

## Abbreviations

PKCε: protein kinase C epsilon
ATF2: activating transcription factor 2
AP1: the activator protein 1
tGCI: transient global cerebral ischemia
ECL: enhanced chemiluminescence
PFA: paraformaldehyde
H&E: hematoxylin and eosin
DAPI: 4′, 6-diamidino-2-phenylindole
XPO1: exportin 1
CRM1: chromosome region maintenance protein 1
LMB: leptomycin B
